# Variations in the alveolar bone morphology in maxillary molar area: a retrospective CBCT study

**DOI:** 10.1186/s12903-024-04588-w

**Published:** 2024-08-01

**Authors:** Yao Tang, Wenhsuan Lu, Yunfan Zhang, Weiqiang Wu, Qiannan Sun, Yuning Zhang, Xiaomo Liu, Wei Liang, Si Chen, Bing Han

**Affiliations:** 1 Department of Orthodontics, Cranial-Facial Growth and Development Center, Peking University School and Hospital of Stomatology, 22 Zhongguancun South Avenue, Haidian District, 100081 Beijing, PR China; 2 National Center for Stomatology & National Clinical Research Center for Oral Diseases & National Engineering Research Center of Oral Biomaterials and Digital Medical Devices & Research Center of Engineering and Technology for Computerized Dentistry Ministry of Health, NMPA Key Laboratory for Dental Materials, Beijing, 100081, PR China; 3 Stomatology Hospital, School of Stomatology Zhejiang Provincial Clinical Research Center for Oral Diseases, Key Laboratory of Oral Biomedical Research of Zhejiang Province, Zhejiang University School of Medicine, Cancer Center of Zhejiang University, Hangzhou, 310006 China

**Keywords:** Cone-beam computed tomography, Temporary anchorage device, Alveolar bone morphology, Maxillary retromolar space

## Abstract

**Background:**

This study quantitatively analyzed the anatomic structure of the alveolar bone in the maxillary molar region at three potential locations for Temporary Anchorage Device (TAD) placement. Additionally, the study compared the variability in this region across different age groups, sagittal skeletal patterns, vertical facial types, and sexes.

**Methods:**

In this retrospective cone-beam computed tomography study, the buccal alveolar bone was analyzed in the posterior molar area of 200 patients, the measurement items include buccal alveolar bone height, alveolar bone thickness, interradicular distance, and maxillary retromolar space.

**Results:**

Buccal alveolar height was greatest in the U56 region. The interradicular space was largest in the U56 region and increased from the alveolar crest to the sinus floor. Buccal alveolar bone thickness was highest in the U67 region and generally increased from the alveolar crest to the sinus floor. The maxillary retromolar space gradually increased from the alveolar crest to the root apex.

**Conclusions:**

TADs are safest when placed in the buccal area between the maxillary second premolar and the first molar, particularly at the 9 mm plane. The U67 region is the optimal safe zone for TAD placement for maxillary dentition distalization. TADs placement in adolescents can be challenging. Maxillary third molar extraction can be considered for maxillary dentition distalization.

**Supplementary Information:**

The online version contains supplementary material available at 10.1186/s12903-024-04588-w.

## Introduction

Anchorage control plays a key role in successful orthodontic treatment, and orthodontists utilize various methods to achieve it in clinical practice. During the past decade, the use of temporary anchorage devices (TADs) to achieve absolute anchorage has been established in clinical orthodontics, being effective in various types of malocclusion [1]. TADs are widely used in clinical practice because of their advantages, including smaller size, simple surgical placement, short or no waiting time, easy removal after treatment, and low cost [2]. Maxillary and mandibular interradicular sites are preferred for TAD placement because of the ease of placement and direct orthodontic force application. However, interradicular TADs may damage the tooth roots at the placement site and interfere with the desired orthodontic tooth movement [3]. Previous studies have reported a failure rate of around 13.5% (95% confidence interval: 11.5–15.9%) for micro-implants, which was related to factors, such as patient age, timing of loading force, and anatomical structure of the implant site [4, 5].

It is important to analyze bone parameters, including the width and height, at the planned insertion site. Previous studies have explored the alveolar bone in maxillary molar region and demonstrated that the distance between the root of the second premolar and the mesial root of the first molar is the largest, while the buccal alveolar bone is the thickest in the region between the maxillary first and second molars. However, these studies did not provide a detailed taxonomic analysis [5–7].

The treatment of Class II malocclusion or bimaxillary protrusion may require the distalization of maxillary molars or even the entire dentition to achieve treatment goals. Distalization of the first molar is difficult after the eruption of the second molar, and often requires the use of TADs. The available maxillary retromolar space determines the extent of distalization.

The primary objective of this study was to quantitatively analyze the width and height of the buccal region at three TAD placement locations in relation to maxillary molars. The study also assessed the retromolar space available for maxillary dentition distalization. Bone parameters were compared across sexes, ages, sagittal skeletal types (Class I–III), and vertical facial types (hypodivergent, normodivergent, and hyperdivergent).

## Materials and methods

Cone-beam computed tomography (CBCT) images of 200 patients were obtained from the archive database of the Peking University School and Hospital of Stomatology, Beijing, China. These patients included 105 adults (20 males aged 20.7 ± 4.2 years and 85 females aged 24.9 ± 6.2 years) and 95 adolescents (38 males aged 13.7 ± 1.8 years and 57 females aged 14.1 ± 2.3 years). CBCT was used to examine the interradicular bone for miniscrew insertion, impacted third molars, and osseous structures for temporomandibular joint and orthognathic surgery. The study was approved by the ethics committee of Peking University Hospital of Stomatology (PKUSSIRB-202,280,126). Written informed consent was obtained from all participants and their parents or guardians.

The exclusion criteria were: (1) those who had maxillary molars extracted; (2) individuals with implants or pontics replacing maxillary molars; (3) CBCT scans showing supernumerary teeth, enlarged cystic follicles, or other pathologies; (4) CBCT scans showing impacted teeth in the region of interest; and (5) patients with periodontal disease, a history of orthodontic treatment or orthognathic surgery, or any genetic syndromes.

The scans were divided into two age groups: adults and adolescents, then further divided into sub-groups based on the skeletal malocclusion type (I, II, and III for ANB angles of 0–5°, > 5°, and < 0°, respectively) and facial type (hypodivergent, normodivergent, and hyperdivergent for MP-SN angles of < 27°, 27–37°, and > 37°, respectively) [3]. CBCT images were obtained using the same machine (NewTom, Verona, Italy) with exposure settings of 110 kV, 0.07 mA, and a 153.6-mm field of view (FOV). After acquisition, raw data were converted into the Digital Imaging and Communications in Medicine (DICOM) format and reconstructed with a voxel size of 0.3 mm^3^. The DICOM data were then saved in a personal computer for analysis using the Mimics Research software (version 20.0, Leuven, Belgium).

**Fig. 1:**
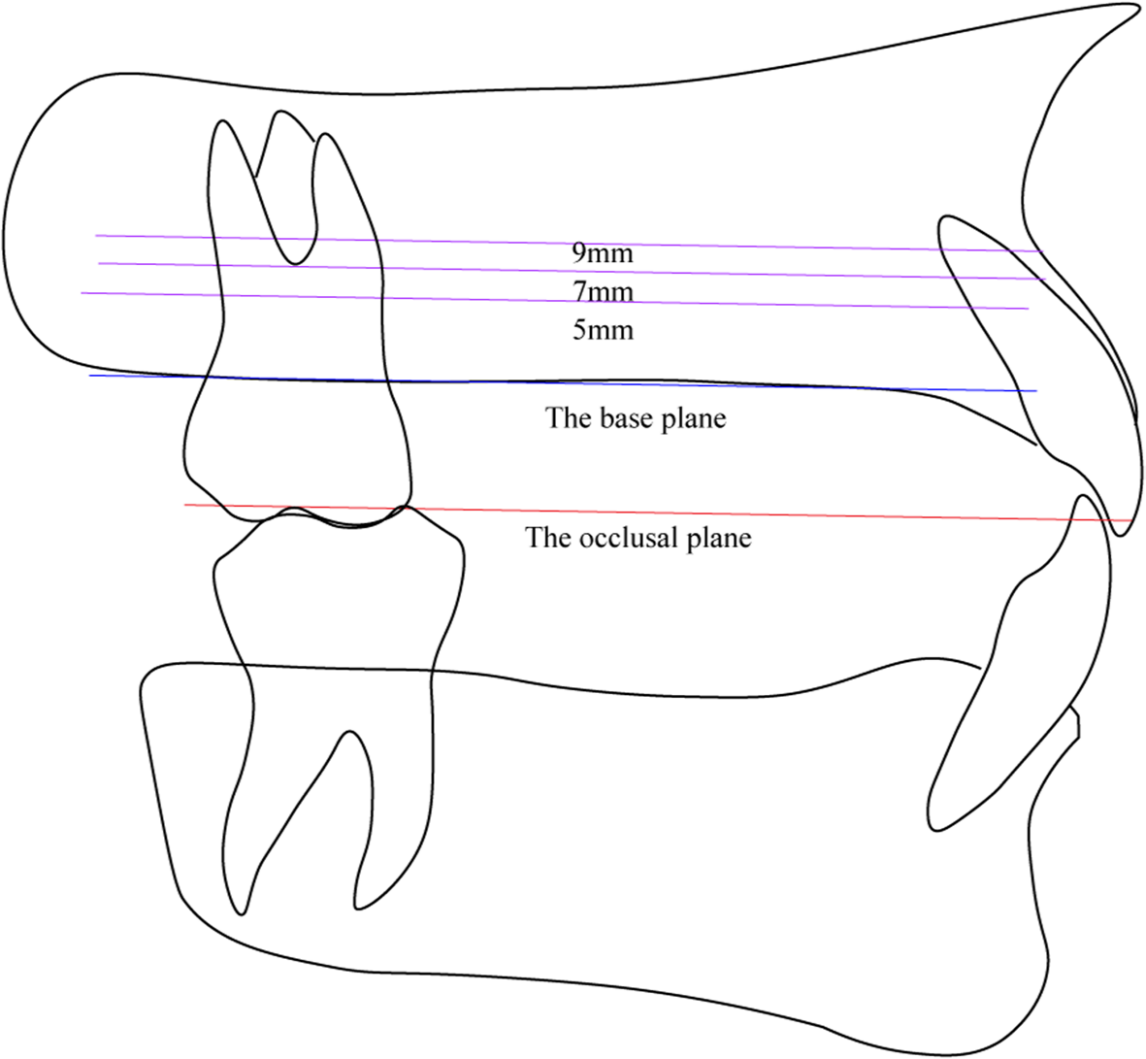
Reference lines for the planes at 5, 7, and 9 mm above the measurement base plane (alveolar crest edge) in the sagittal view

The reference plane for measurement was the occlusal plane through the midpoints of incisors and mesiobuccal cusps of both first molars. A plane parallel to the occlusal plane, passing through the alveolar ridge, was taken as the base plane (Fig. 1). Three areas were measured in this study: between the maxillary second premolar and the first molar (U56), between the mesiobuccal and distobuccal roots of the first molar (U6md), and between the first and second molar (U67).

**Fig. 2:**
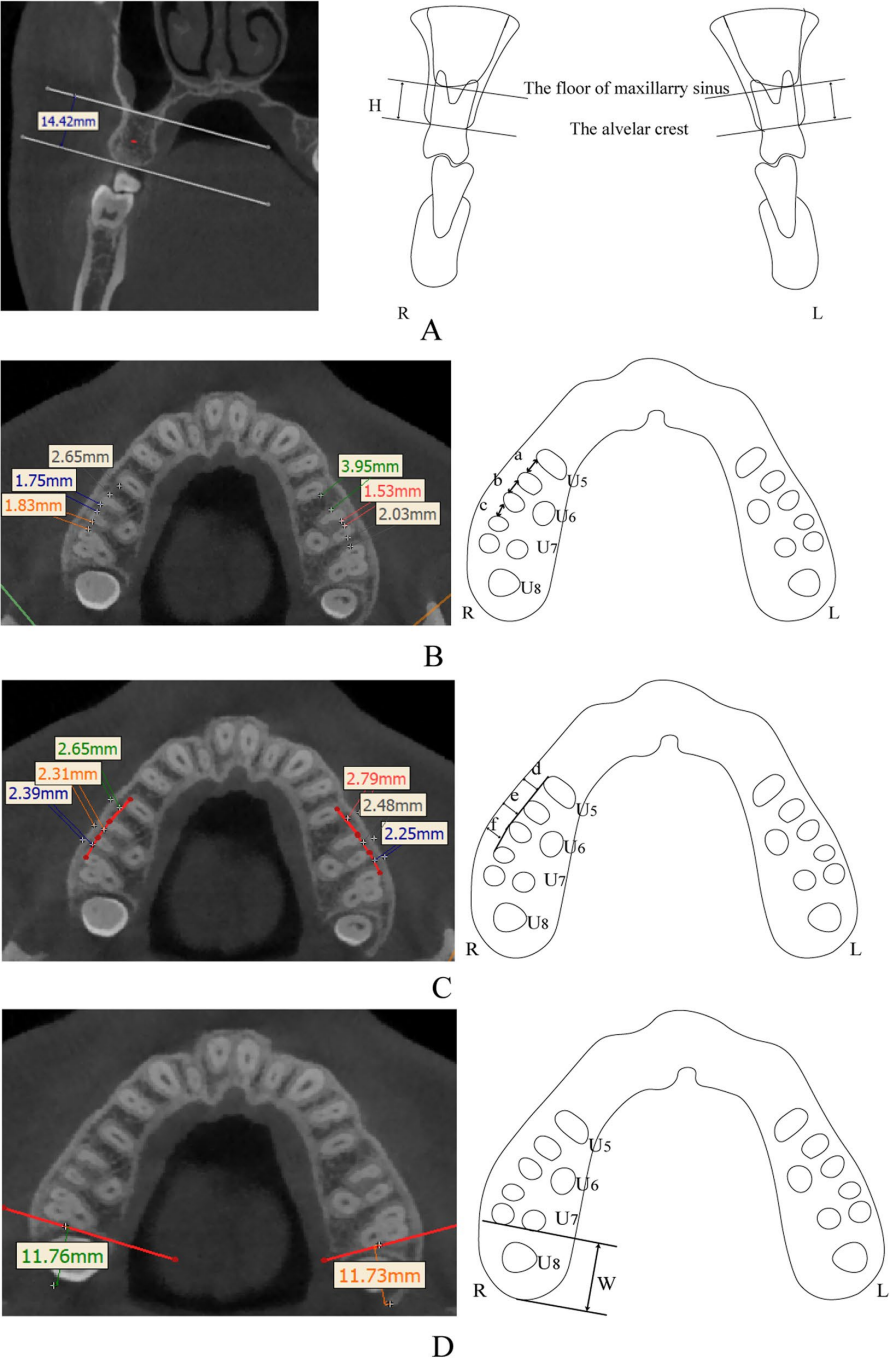
**A** Measurement of the distance between the alveolar edge of the crest and the sinus floor at the buccal side in the middle of U56, U6md, and U67. L: left, R: right, H: buccal bone height. **B** buccal interradicular distance in axial images. a, b, c: buccal interradicular distances for U56, U6md, and U67, respectively. **C** Measurement of buccal bone thickness, d, e, f: buccal alveolar bone thickness for U56, U6md, and U67, respectively. **D** Measurement of the maxillary retromolar space, W: maxillary retromolar space

In the middle of U56, U6md, and U67, the maxillary buccal alveolar bone height (BAH) was measured from the maxillary sinus floor to the buccal alveolar ridge edge in coronal sections (Fig. 2A). The buccal interradicular distance (BID, the shortest distance between adjacent roots), buccal alveolar bone thickness (BAT, the distance from a tangent to adjacent roots to the buccal alveolar bone surface), and maxillary retromolar space (MRS, the distance from distal surface of second molar to the posterior edge of maxilla) were measured at planes set at 5, 7, and 9 mm above the alveolar ridge (Figs. 1 and 2B and D) [7].

Data measurement and analysis were performed by a single examiner. A random sample of 30 was analyzed twice at 2-week intervals by the same examiner. Reliability was assessed by measuring the intraclass correlation coefficients. All the values were > 0.80 (Table S1), indicating that the measurements were reliable (*P* > 0.05).

### Statistical analysis

SPSS Statistics software (version 20.0; IBM Corp., Armonk, NY) was used for statistical analyses. Descriptive statistics were analyzed for all parameters. Normality of the parameters was assessed using the Kolmogorov-Smirnov test. Independent sample t test or Mann-Whitney U test was used to compare the parameters between adult and juvenile groups and between males and females. One way analysis of variance (ANOVA) or Kruskal-Wallis test was used to compare the parameters among the sagittal skeletal types and facial types. The significance level was set at *P* < 0.05.

## Results

Table 1 presents descriptive statistics for the BAH across three distinct anatomical sites. In the U56 region, the BAH was 9.93 ± 3.78 mm, which was significantly greater than that for U6md (8.01 ± 2.71 mm) and U67 (8.18 ± 2.56 mm). The comparison between males and females revealed no significant differences in BAH (Table S3). However, different age groups exhibited variations, with the BAH in the adolescent group being consistently lower than that in the adult group for all three regions (Table 2). Among different sagittal skeletal groups, Class I individuals had the lowest BAH in the U56 area (Table S4). Furthermore, the BAH for hypodivergent participants was significantly greater than that for hyperdivergent participants in the U67 area (Table S5).

**Table 1:** Buccal alveolar bone heights (mm) at the three anatomical sites

Area	Mean	SD	Min	Max	P25	P50	P75	H	*P*
U56^a^	9.93	3.78	1.66	22.50	7.19	9.31	11.87	70.05	< 0.001***
U6md^b^	8.01	2.71	1.71	20.56	6.28	7.68	9.19		
U67^b^	8.18	2.56	1.91	17.54	6.38	7.90	9.70		

*SD* standard deviation, Kruskal-Wallis test

U56, between the maxillary second premolar and the first molar

U6md, between the mesiobuccal and distobuccal roots of the first molar

U67, between the first and second molar

^a,b^statistically significant differences were observed between groups marked with the different letter

***Statistically significant at *P* <0.001

**Table 2:** Comparison of buccal alveolar bone height (mm) of different ages

Area	Adult				Adolescent				Z	*P*
	Mean	SD	Min	Max	Mean	SD	Min	Max		
U56	10.90	4.19	1.66	22.50	8.88	2.94	2.78	19.53	-4.86	< 0.001***
U6md	8.46	3.10	1.71	20.56	7.53	2.10	2.09	13.53	-2.58	0.010*
U67	8.39	2.86	1.91	17.54	7.96	2.17	2.48	13.63	-1.09	0.277

*SD* standard deviation, Mann-Whitney U test

U56, between the maxillary second premolar and the first molar

U6md, between the mesiobuccal and distobuccal roots of the first molar

U67, between the first and second molar

*Statistically significant at *P* <0 .05

***Statistically significant at *P* <0 .001

**Fig. 3:**
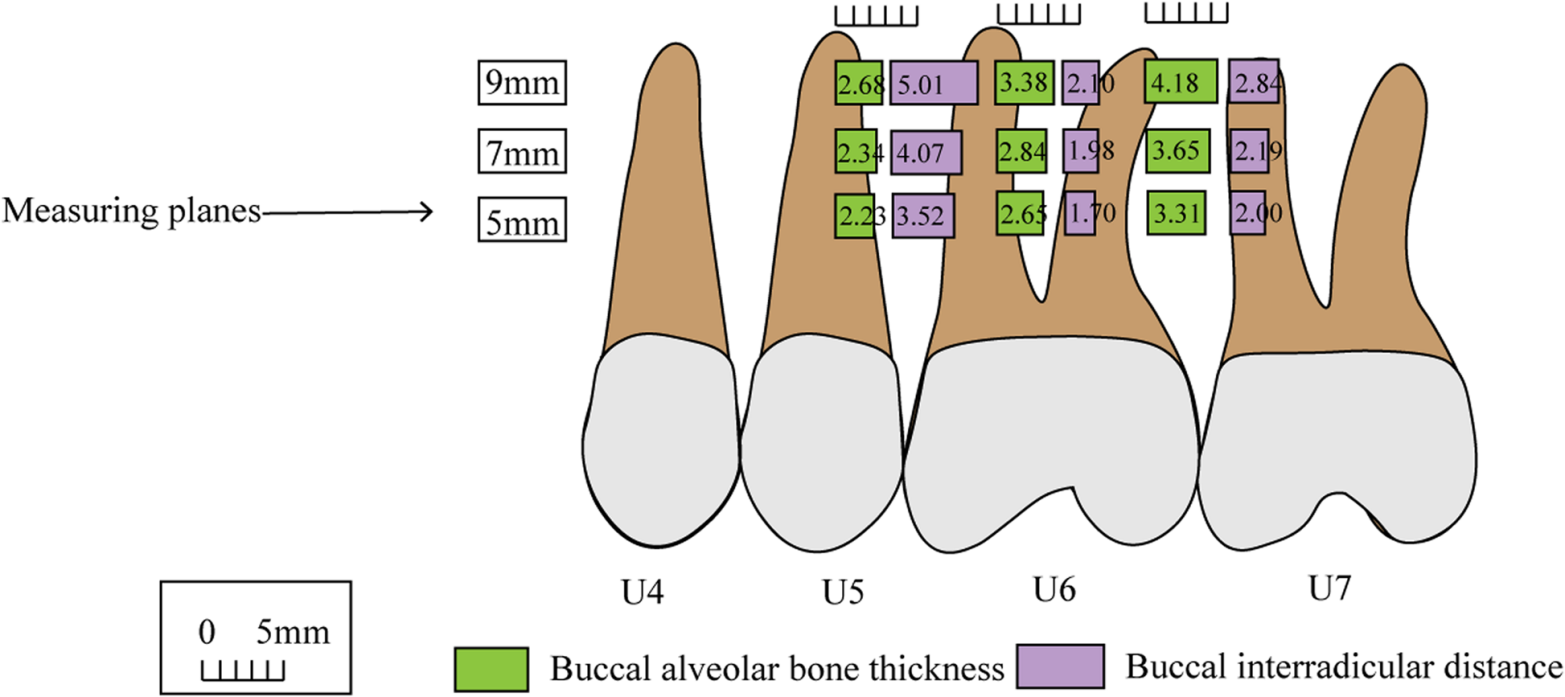
Schematic diagram of buccal alveolar bone thickness and interradicular distance in different regions

Table 3 presents descriptive statistics for BAT across various regions and planes. The BAT exhibited an increase at the 5, 7, and 9 mm planes, with the U67 region displaying the greatest thickness, followed by the U6md region, while the U56 region showed the smallest thickness (Fig. 3, Table S6). A comparison between males and females showed no significant differences across different regions (Table S7). Meanwhile, the BAT in the adolescent group was found to be greater than that in the adult group (Table 4). When comparing different sagittal skeletal types, the BAT in Class I was greater than that in Class III (Table S8). Among the various vertical facial types, the normodivergent group had the highest BAT, but the differences among these groups were not statistically significant (Table S9).

**Table 3:** Buccal alveolar bone thickness (mm) in different regions

Area	Plane	Mean	SD	Min	Max	Percentile			F/H	*P*
						25^th^	50^th^	75^th^		
U56	5 mm^a^	2.23	0.91	0.34	6.04	1.53	2.11	2.88	14.25	< 0.001^K^***
	7 mm^a^	2.34	1.10	0.28	6.38	1.55	2.18	3.09		
	9 mm^b^	2.68	1.47	0.35	8.62	1.66	2.42	3.47		
U6md	5 mm^a^	2.65	1.05	0.52	7.44	1.87	2.56	3.31	32.88	< 0.001^K^***
	7 mm^a^	2.84	1.29	0.41	7.80	1.91	2.69	3.68		
	9 mm^b^	3.38	1.72	0.40	9.34	2.07	3.09	4.47		
U67	5 mm^a^	3.31	0.85	1.07	6.57	2.71	3.26	3.82	66.87	< 0.001^A^***
	7 mm^b^	3.65	0.99	0.69	7.21	3.03	3.61	4.25		
	9 mm^c^	4.18	1.29	0.76	8.17	3.28	4.08	4.93		

*SD* standard deviation, *K* Kruskal-Wallis test, *A* One-way ANOVA

U56, between the maxillary second premolar and the first molar

U6md, between the mesiobuccal and distobuccal roots of the first molar

U67, between the first and second molar

^a,b,c^statistically significant differences observed between groups marked with the different letter

*** Statistically significant at *P*<0.01

**Table 4:** Buccal alveolar bone thickness (mm) of different ages

Area	Plane	Adult				Adolescent				t/Z	*P*
		Mean	SD	Min	Max	Mean	SD	Min	Max		
U56	5 mm	1.97	0.86	0.51	6.04	2.51	0.89	0.34	5.05	-6.360	< 0.001^U^***
	7 mm	2.03	1.05	0.50	6.38	2.68	1.06	0.28	6.37	-6.449	< 0.001^U^***
	9 mm	2.21	1.34	0.35	8.62	3.16	1.45	0.39	8.61	-6.786	< 0.001^U^***
U6md	5 mm	2.28	0.98	0.52	7.44	3.06	0.97	0.87	6.21	-8.185	< 0.001^U^***
	7 mm	2.34	1.15	0.41	7.80	3.38	1.23	0.53	7.60	-8.572	< 0.001^U^***
	9 mm	2.62	1.41	0.55	9.34	4.14	1.66	0.40	9.01	-8.855	< 0.001^U^***
U67	5 mm	3.21	0.88	1.07	6.00	3.41	0.79	1.34	6.57	2.426	0.016^T^***
	7 mm	3.46	1.04	0.69	7.07	3.86	0.89	1.40	7.21	4.174	< 0.001^T^***
	9 mm	3.82	1.23	1.33	8.17	4.55	1.24	0.76	7.74	5.697	< 0.001^T^***

*SD* standard deviation, *U* Mann-Whitney U test, *T* Independent sample t test

U56, between the maxillary second premolar and the first molar

U6md, between the mesiobuccal and distobuccal roots of the first molar

U67, between the first and second molar

***Statistically significant at *P* <0 .001

Table 5 presents descriptive statistics for BID across various regions and planes. The BID increased at the 5, 7, and 9 mm planes, with the largest distance observed in the U56 region, followed by the U67 region, and the smallest distance recorded in the U6md region (Fig. 3, Table S10). Compared to males, the BID was larger among females in the U67 region, but smaller in the U6md region (Table S11). Compared to the adolescent group, the adult group had larger BID at the 5, 7, and 9 mm planes in the U67 region, and smaller BID at the 5 mm plane in the U56 region (Table 6). Among different sagittal skeletal types, the BID in Class I individuals was the largest in the U56 and U6md regions, but the smallest in the U67 region (Table S12). The differences among different vertical facial types were not statistically significant (Table S13).

**Table 5:** Buccal interradicular distance (mm) at different regions

Area	Plane	Mean	SD	Min	Max	Percentiles			F/H	*P*
						25th	50th	75th		
U56	5 mm^a^	3.52	0.98	0.89	6.26	2.83	3.56	4.22	149.15	< 0.001^A^***
	7 mm^b^	4.07	1.15	1.38	7.26	3.22	4.10	4.88		
	9 mm^c^	5.01	1.44	1.43	9.37	4.00	5.02	5.95		
U6md	5 mm^a^	1.70	0.59	0.35	3.82	1.29	1.68	2.08	35.90	< 0.001^K^***
	7 mm^b^	1.98	0.74	0.41	4.33	1.46	1.92	2.46		
	9 mm^b^	2.10	1.05	0.37	8.12	1.26	1.97	2.71		
U67	5 mm^a^	2.00	1.08	0.03	10.68	1.27	1.83	2.58	69.90	< 0.001^K^***
	7 mm^a^	2.19	1.26	0.26	7.52	1.23	1.97	2.92		
	9 mm^b^	2.84	1.52	0.38	8.55	1.67	2.64	3.86		

*SD* standard deviation, *K* Kruskal-Wallis test, *A* One-way ANOVA

U56, between the maxillary second premolar and the first molar

U6md, between the mesiobuccal and distobuccal roots of the first molar

U67, between the first and second molar

^a,b,c^statistically significant differences observed between groups marked with the different letter

***Statistically significant at *P* <0 .001

**Table 6:** Buccal interdental root distances (mm) of different ages

Area	Plane	Adult				Adolescent					t/Z		*P*
		Mean	SD	Min	Max	Mead	SD	Min	Max				
U56	5 mm	3.41	0.99	0.89	5.87	3.64	0.97	1.30	6.26		2.365		0.018^T^*
	7 mm	4.02	1.17	1.38	7.14	4.13	1.12	1.41	7.26		0.899		0.369^T^
	9 mm	4.98	1.50	1.52	9.37	5.03	1.38	1.43	8.81		0.309		0.758^T^
U6md	5 mm	1.77	0.60	0.55	3.54	1.63	0.57	0.35	3.82		-2.476		0.014 ^T^*
	7 mm	1.93	0.74	0.41	3.94	2.04	0.74	0.44	4.33		1.538		0.125^T^
	9 mm	1.97	0.96	0.41	5.35	2.23	1.12	0.37	8.12		-2.265		0.023^U^*
U67	5 mm	2.28	1.18	0.36	10.68	1.68	0.87	0.03	6.08		-5.896		< 0.001^U^***
	7 mm	2.66	1.29	0.52	6.61	1.67	0.98	0.26	7.52		-8.250		< 0.001^U^***
	9 mm	3.56	1.53	0.39	8.55	2.11	1.12	0.38	8.48		-9.239		< 0.001^U^***

*SD* standard deviation, *U* Mann-Whitney U test, *T* Independent sample t test

U56, between the maxillary second premolar and the first molar

U6md, between the mesiobuccal and distobuccal roots of the first molar

U67, between the first and second molar

*Statistically significant at *P* <0 .05

***Statistically significant at *P* <0 .001

Table 7 provides descriptive statistics for the MRS at the 5, 7, and 9 mm planes. The MRS exhibited a sequential increase with increasing plane distance. This progression was statistically significant. A comparison between males and females revealed no significant differences (Table S14). In terms of age, the MRS in the adolescent group was consistently smaller than that in the adult group at all three planes (Table 8). Within different sagittal skeletal groups, Class I individuals exhibited a smaller MRS than those in the remaining two groups (Table S15). However, when comparing different vertical facial types, there were no significant differences (Table S16). Furthermore, compared to individuals with congenitally missing maxillary third molars, those without this condition displayed a wider MRS, with statistically significant differences noted at the 7 and 9 mm planes (Table S17).

**Table 7:** Maxillary retromolar space (mm) at the 5, 7, and 9 mm planes

Plane	Mean	SD	Min	Max	Percentiles			H	*P*
					25th	50th	75th		
5 mm^a^	9.33	2.70	1.75	17.84	7.50	9.63	11.20	84.34	< 0.001***
7 mm^b^	10.29	2.51	3.20	17.85	8.70	10.45	11.94		
9 mm^c^	11.08	2.41	2.15	18.40	9.65	11.23	12.60		

*SD* standard deviation, Kruskal-Wallis test

^a,b,c^statistically significant differences observed between groups marked with the different letter

***Statistically significant at *P* <0 .001

**Table 8:** Comparison of maxillary retromolar space (mm) of different ages

Plane	Adult				Adolescent				t/Z	*P*
	Mean	SD	Min	Max	Mean	SD	Min	Max		
5 mm	10.23	2.28	3.16	17.84	8.35	2.78	1.75	15.99	-6.65	< 0.001^U^***
7 mm	10.96	2.11	4.79	17.85	9.56	2.70	3.2	16.53	-5.40	< 0.001^U^***
9 mm	11.50	2.25	2.15	18.40	10.63	2.50	3.64	18.17	-3.65	< 0.001^T^***

*SD* standard deviation, *U* Mann-Whitney U test, *T* Independent sample t test

***Statistically significant at *P* <0 .001

## Discussion

Compared with CT, CBCT has gained popularity due to lower radiation exposure and higher definition, becoming a widely used tool for both qualitative and quantitative assessment of alveolar bone conditions [8]. Previous studies have demonstrated that CBCT is comparable to direct measurements in terms of bone height and thickness, and it offers significantly higher accuracy than traditional 2D radiography [9–11].

In this study, we conducted detailed measurements of the most commonly used areas for TAD placement based on a large sample of CBCT data. The results indicated that the BAH in the U56 area was significantly greater than in the U6md and U67 areas. The BAH in the adult group exceeded that in the adolescent group, Liu et al. also reported greater BAH in the U56 region for adults [7], owing to continuous growth of the jawbone, the BAH gradually increases from adolescents to adults [12]. In terms of vertical facial types, our study revealed that BAH in the hyperdivergent group was smaller than in the hypodivergent group, consistent with results from Swasty et al. [13] Xin et al. found that implanting miniscrews at a 45° angle to the long axis of the teeth can enhance stability by increasing the contact area between miniscrews and the bone cortex, although it increases the risk of maxillary sinus perforation [5]. In our study, the BAH in the U56 region ranged from 2.78 to 22.79 mm, demonstrating significant individual variations. This highlights the importance of CBCT measurements when selecting TAD lengths, particularly for adolescents.

Currently, the diameter of commonly used TADs ranges from 1.2 mm to 2 mm [14]. Since TADs often remain in the bone for over 6 months, some experts recommend using TADs with a maximum diameter of 2 mm to ensure sufficient strength [7]. Considering the necessary safe distance of at least 0.5 mm between the miniscrew and adjacent tissues, a 2-mm diameter miniscrew can be safely used when the interradicular space exceeds 3 mm [15]. According to our results, the mean BID was greater than 3 mm at the 5, 7, and 9 mm planes for the U56 region and at the 9 mm plane for the U67 region. Additionally, the BID tended to increase from the crest edge to the sinus floor. Therefore, a 2 mm screw may be used without issues in most patients. But there are patients with BID values below 3 mm, then smaller diameter screws may be more appropriate.

Given that the BAH in the U56 region is the highest among the three measurement areas, it is safer to implant TADs in this area, which is consistent with prior studies [5, 6]. Additionally, compared to adults, the adolescent group displayed a larger BID at the 5 mm plane in the U56 area, but a smaller BID at the 5, 7, and 9 mm planes in the U67 area where it was only about 2 mm. This suggests that placing TADs between the maxillary second premolar and the first molar is more advisable for adolescents. When assessing different sagittal skeletal groups, it was observed that the BID in the U56 and U6md regions of skeletal Class I individuals was greater than in skeletal Class II and III individuals, despite the BAH being smaller in Class I compared to Class II and III. Regarding vertical skeletal groups, the BAH and the BID in the U56 area of hypodivergent subjects were both greater than those in hyperdivergent subjects, indicating that the risk of root damage and maxillary sinus perforation is higher in hyperdivergent individuals.

To prevent interferences with tooth movement during maxillary molar distalization, some studies have proposed placing TADs in the buccal alveolar bone in the infrazygomatic crest region [16]. This study found that BAT in the maxillary molar area increased progressively at 5, 7, and 9 mm planes, with the greatest thickness in the U67 area, followed by the U6mm area, while the U56 area had the smallest thickness. To ensure periodontal health, a minimum of 1 mm of alveolar bone should surround the screw when placing TADs on the buccal side of the teeth [14]. In this study, the average BAT in the U67 area exceeded 3 mm at all three measured planes. Therefore, placing a 2-mm diameter TAD in this area is feasible. Liou et al. suggested that the depth of implantation should be at least 6 mm to maintain TAD stability, although increasing the TAD lengths also increases the risk of maxillary sinus injury [17]. Ardekian et al. reported that perforations less than 2 mm in the maxillary sinus often heal spontaneously and rarely result in complications [18]. Moreover, piercing both cortical plates (maxillary sinus floor and buccal cortical plate) can provide bicortical miniscrew anchorage, which has been shown to be superior to monocortical anchorage in terms of resistance to miniscrew movement [5, 19]. Therefore, in clinical practice, TAD lengths should be chosen according to specific needs.

The extent of maxillary dentition distalization is limited by the dimensions of the MRS. This study found that the MRS increased progressively at 5, 7, and 9 mm planes, indicating that maxillary dentition distalization is restricted by the distal alveolar bone in the cervical plane of the second molar. Measurements in the adolescent group were smaller than those in the adult group, suggesting that maxillary molar distalization may be less effective in adolescents than in adults, which could be attributed to jawbone growth [12]. In terms of sagittal classification, the MRS in skeletal Class I individuals was smaller than that in skeletal Class II and III individuals. Furthermore, individuals with congenital loss of maxillary third molars exhibited smaller MRS compared to those with impacted or erupted third molars, a finding in line with Mah et al.’s study [20]. Therefore, extracting the third molar to create more space for dentition distalization may be a viable option.

Females are more concerned about the esthetics of their teeth than males, leading to a more active demand for treatment in females [21]. In our study, a total of 200 samples were included, due to the significantly lower proportion of males in orthodontic patients, the gender ratio of the samples is not ideal (males/females:142/58), which could have influenced the difference in measurements by sex. And we mainly focused on the relationship between alveolar bone and teeth, did not consider anatomical variants in the maxillary sinus floor as variable factors, which is a limitation of our study. In the future, we plan to discuss anatomical variations of maxillary sinus floor in our upcoming new research., as well balance the gender ratio of the sample.

## Conclusions

1. The maximum space was observed to be at 9mm plane apically from alveolar crest between max first molar and second molar which would make this site a safer location for the insertion of the TAD.
2. The region between the maxillary first and second molars has the greatest BAH and is, therefore, the safest zone for TAD placement for maxillary dentition distalization.
3. Compared to adults, the alveolar bone thickness, height, and retromolar space is smaller in adolescents, which might increase the difficulty in TAD placement and dentition distalization.
4. Compared to hypodivergent subjects, the BAH and the BID is smaller in hyperdivergent subjects, which might increase the risk of root damage and maxillary sinus perforation in TAD placement.
5. Individuals with congenital loss of maxillary third molars exhibited smaller retromolar space compared to those with impacted or erupted third molars, it may be a viable option to create more space for dentition distalization by extracting the third molar.

### Supplementary Information

Supplementary Material 1.

## Data Availability

Data is provided within the manuscript or supplementary information files. The data sets used and/or analysed during the current study are available from the corresponding author on reasonable request.
