# Supplementary material for: Variations in the alveolar bone morphology in maxillary molar area: a retrospective CBCT study
Source: BMC Oral Health. 2024 Aug 1;24:872. doi: 10.1186/s12903-024-04588-w (PMC11295338; doi:10.1186/s12903-024-04588-w)
Supplement: Supplementary file 1 — Supplementary Material 1. [file 12903_2024_4588_MOESM1_ESM.doc]

Supporting Information

**Variability of alveolar bone morphology in maxillary molar area: a retrospective CBCT study**

*Yao Tang***^‡^***, Wenhsuan Lu* **^‡^***, Yunfan Zhang***^‡^***, Weiqiang Wu, Qiannan Sun, Yuning Zhang, Xiaomo Liu, Wei Liang*, Si Chen*, and Bing Han**

**Table of contents**

[Repeated measurement stability analysis 3](#__RefHeading___Toc117585853)

[Table S1-S17 4](#__RefHeading___Toc117585853)

**Repeated measurement stability analysis**

Data measurement and analysis were performed by a single examiner. A random sample of 30 was analyzed twice at 2-week intervals by the same examiner. Reliability was assessed by measuring the intraclass correlation coefficients; the values were > 0.80 (Table S1), indicating that the measurements were reliable (P > 0.05).

**Table S1-S17**

**Table S1**. Repeated measurement stability analysis.

| Item | Area | Plane | ICC | 95%CI |
| --- | --- | --- | --- | --- |
| Buccal alveolar bone height | U56 |  | 0.988 | （0.974，0.995） |
|  | U6md |  | 0.976 | （0.944，0.990） |
|  | U67 |  | 0.963 | （0.919，0.984） |
| Maxillary retromolar space | U56 |  | 0.906 | （0.790，0.958） |
|  | U6md |  | 0.912 | （0.811，0.960） |
|  | U67 |  | 0.911 | （0.748，0.965） |
| Buccal alveolar bone thickness | U56 | 5mm | 0.944 | （0.879，0.975） |
|  |  | 7mm | 0.917 | （0.820，0.963） |
|  |  | 9mm | 0.884 | （0.748，0.949） |
|  | U6md | 5mm | 0.865 | （0.700，0.940） |
|  |  | 7mm | 0.898 | （0.782，0.954） |
|  |  | 9mm | 0.894 | （0.768，0.954） |
|  | U67 | 5mm | 0.884 | （0.758，0.947） |
|  |  | 7mm | 0.942 | （0.874，0.974） |
|  |  | 9mm | 0.940 | （0.864，0.974） |
| Buccal interradicular distance | U56 | 5mm | 0.920 | （0.824，0.965） |
|  |  | 7mm | 0.910 | （0.804，0.960） |
|  |  | 9mm | 0.880 | （0.733，0.948） |
|  | U6md | 5mm | 0.877 | （0.742，0.943） |
|  |  | 7mm | 0.910 | （0.807，0.959） |
|  |  | 9mm | 0.944 | （0.866，0.977） |
|  | U67 | 5mm | 0.953 | （0.896，0.979） |
|  |  | 7mm | 0.940 | （0.868，0.973） |
|  |  | 9mm | 0.887 | （0.770，0.949） |

ICC, Intraclass Correlation Coefficient; CI, Confidence Internal.

U56, between the maxillary second premolar and the first molar

U6md, between the mesiobuccal and distobuccal roots of the first molar

U67, between the first and second molar

**Table S2**. Sample distribution for skeletal type (Cl Ⅰ-Ⅱ-Ⅲ) and facial type (hypo-normal and hyper divergent)

| Facial type  Skeletal type | hyper | normal | hypo | total |
| --- | --- | --- | --- | --- |
| Ⅰ | 17 | 48 | 17 | 82 |
| Ⅱ | 25 | 33 | 10 | 68 |
| Ⅲ | 16 | 22 | 12 | 50 |
| total | 58 | 103 | 39 | 200 |

**Table S3**. Comparison of buccal alveolar bone height (mm) of different genders

| Area | Male | | Female | | Z | P |
| --- | --- | --- | --- | --- | --- | --- |
|  | Mean | SD | Mean | SD |  |  |
| U56 | 9.82 | 3.60 | 9.98 | 5.85 | -0.420 | 0.675 |
| U6md | 8.02 | 2.67 | 8.01 | 2.73 | -0.077 | 0.939 |
| U67 | 8.52 | 2.79 | 8.05 | 2.45 | -1.089 | 0.276 |

SD: standard deviation; Mann-Whitney U test;

U56, between the maxillary second premolar and the first molar

U6md, between the mesiobuccal and distobuccal roots of the first molar

U67, between the first and second molar

**Table S4.** Comparison of buccal alveolar bone height (mm) of different sagittal skeletal types.

| Area | Sagittal skeletal type | Mean | SD | Percentiles | | | H | P |
| --- | --- | --- | --- | --- | --- | --- | --- | --- |
|  |  |  |  | 25 | 50 | 75 |  |  |
| U56 | Ⅰ ^a^ | 9.23 | 3.53 | 6.67 | 8.70 | 10.61 | 12.91 | 0.002** |
|  | Ⅱ ^b^ | 10.18 | 3.54 | 7.83 | 9.64 | 12.24 |  |  |
|  | Ⅲ ^b^ | 10.75 | 4.28 | 7.30 | 10.38 | 13.66 |  |  |
| U6md | Ⅰ | 7.65 | 2.51 | 6.01 | 7.43 | 8.60 | 4.62 | 0.100 |
|  | Ⅱ | 8.17 | 2.60 | 6.49 | 7.77 | 9.48 |  |  |
|  | Ⅲ | 8.42 | 3.09 | 6.34 | 7.88 | 10.22 |  |  |
| U67 | Ⅰ | 7.99 | 2.48 | 6.43 | 7.72 | 9.54 | 4.60 | 0.100 |
|  | Ⅱ | 8.11 | 2.60 | 6.24 | 7.89 | 9.70 |  |  |
|  | Ⅲ | 8.59 | 2.61 | 6.63 | 8.60 | 10.18 |  |  |

SD: standard deviation; Kruskal-Wallis test;

** Statistically significant at P <0 .01.

U56, between the maxillary second premolar and the first molar

U6md, between the mesiobuccal and distobuccal roots of the first molar

U67, between the first and second molar

^a b^ statistically significant differences were observed between groups marked with the different letter.

**Table S5**. Comparison of buccal alveolar bone height (mm) of different vertical facial types.

| Area | Vertical facial type | Mean | SD | Percentiles | | | H | P |
| --- | --- | --- | --- | --- | --- | --- | --- | --- |
|  |  |  |  | 25 | 50 | 75 |  |  |
| U56 | hyperdivergent | 9.87 | 3.66 | 7.24 | 9.43 | 11.53 | 0.18 | 0.916 |
|  | normodivergent | 9.97 | 3.85 | 7.19 | 9.52 | 12.25 |  |  |
|  | hypodivergent | 9.95 | 3.79 | 7.02 | 9.06 | 12.39 |  |  |
| U6md | hyperdivergent | 7.74 | 2.87 | 5.79 | 7.44 | 8.55 | 4.24 | 0.120 |
|  | normodivergent | 8.04 | 2.68 | 6.59 | 7.91 | 9.76 |  |  |
|  | hypodivergent | 8.34 | 2.54 | 6.71 | 7.94 | 9.27 |  |  |
| U67 | Hyperdivergent ^a^ | 7.76 | 2.50 | 5.98 | 7.36 | 9.41a | 8.21 | 0.016* |
|  | Normodivergent ^ab^ | 8.18 | 2.54 | 6.41 | 7.72 | 9.69ab |  |  |
|  | Hypodivergent ^b^ | 8.81 | 2.60 | 6.75 | 8.46 | 9.84b |  |  |

SD: standard deviation; Kruskal-Wallis test;

* Statistically significant at P <0 .05.

U56, between the maxillary second premolar and the first molar

U6md, between the mesiobuccal and distobuccal roots of the first molar

U67, between the first and second molar

^a b^ statistically significant differences were observed between groups marked with the different letter.

**Table S6**. Buccal alveolar bone thickness (mm) in different planes.

| Area | Plane | Mean | SD | Min | Max | Percentile | | | F/H | P |
| --- | --- | --- | --- | --- | --- | --- | --- | --- | --- | --- |
|  |  |  |  |  |  | 25^th^ | 50^th^ | 75^th^ |  |  |
| 5mm | U56^a^ | 2.23 | 0.91 | 0.34 | 6.04 | 1.53 | 2.11 | 2.88 | 238.11 | <0.001^K^*** |
|  | U6md^b^ | 2.65 | 1.05 | 0.52 | 7.44 | 1.87 | 2.56 | 3.31 |  |  |
|  | U67^c^ | 3.31 | 0.85 | 1.07 | 6.57 | 2.71 | 3.26 | 3.82 |  |  |
| 7mm | 5 mm^a^ | 2.34 | 1.10 | 0.28 | 6.38 | 1.55 | 2.18 | 3.09 | 244.62 | <0.001^K^*** |
|  | 7 mm^a^ | 2.84 | 1.29 | 0.41 | 7.80 | 1.91 | 2.69 | 3.68 |  |  |
|  | 9 mm^b^ | 3.65 | 0.99 | 0.69 | 7.21 | 3.03 | 3.61 | 4.25 |  |  |
| 9mm | 5 mm^a^ | 2.68 | 1.47 | 0.35 | 8.62 | 1.66 | 2.42 | 3.47 | 185.41 | <0.001^A^*** |
|  | 7 mm^b^ | 3.38 | 1.72 | 0.40 | 9.34 | 2.07 | 3.09 | 4.47 |  |  |
|  | 9 mm^c^ | 4.18 | 1.29 | 0.76 | 8.17 | 3.28 | 4.08 | 4.93 |  |  |

SD: standard deviation; K: Kruskal-Wallis test; A: One-way ANOVA;

***Statistically significant at P <0 .001.

U56, between the maxillary second premolar and the first molar

U6md, between the mesiobuccal and distobuccal roots of the first molar

U67, between the first and second molar

^a,b,c^ statistically significant differences observed between groups marked with the different letter.

**Table S7.** Comparison of buccal alveolar bone thickness (mm) of different genders.

| Area | Plane | Male | | Female | | t/Z | P |
| --- | --- | --- | --- | --- | --- | --- | --- |
|  |  | Mean | SD | Mean | SD |  |  |
| U56 | 5mm | 2.28 | 0.96 | 2.21 | 0.89 | -0.78 | 0.438 ^U^ |
|  | 7mm | 2.37 | 1.09 | 2.33 | 1.11 | -0.44 | 0.657 ^U^ |
|  | 9mm | 2.69 | 1.40 | 2.68 | 1.51 | -0.33 | 0.741 ^U^ |
| U6md | 5mm | 2.75 | 1.10 | 2.61 | 1.03 | -1.12 | 0.263 ^U^ |
|  | 7mm | 2.87 | 1.31 | 2.82 | 1.29 | -0.34 | 0.737 ^U^ |
|  | 9mm | 3.35 | 1.67 | 3.40 | 1.74 | -0.22 | 0.829^U^ |
| U67 | 5mm | 3.41 | 0.93 | 3.26 | 0.81 | -1.49 | 0.139 ^T^ |
|  | 7mm | 3.72 | 1.00 | 3.62 | 0.99 | -0.91 | 0.364 ^T^ |
|  | 9mm | 4.17 | 1.21 | 4.19 | 1.32 | 0.13 | 0.897^T^ |

SD: standard deviation; U, Mann-Whitney U test; T, Independent sample t test;

U56, between the maxillary second premolar and the first molar

U6md, between the mesiobuccal and distobuccal roots of the first molar

U67, between the first and second molar

**Table S8.** Comparison of buccal bone thickness (mm) among different sagittal skeletal types.

| Area | Plane | Sagittal skeletal type | Mean | SD | Percentiles | | | F/H | P |
| --- | --- | --- | --- | --- | --- | --- | --- | --- | --- |
|  |  |  |  |  | 25 | 50 | 75 |  |  |
| U56 | 5mm | Ⅰ | 2.31 | 0.92 | 1.61 | 2.21 | 3.06 | 4.490 | 0.106 ^K^ |
|  |  | Ⅱ | 2.26 | 0.95 | 1.56 | 2.09 | 2.93 |  |  |
|  |  | Ⅲ | 2.06 | 0.84 | 1.44 | 1.99 | 2.56 |  |  |
|  | 7mm | Ⅰ | 2.47 | 1.18 | 1.49 | 2.32 | 3.29 | 3.223 | 0.200 ^K^ |
|  |  | Ⅱ | 2.29 | 1.10 | 1.59 | 2.12 | 2.98 |  |  |
|  |  | Ⅲ | 2.21 | 0.96 | 1.36 | 2.06 | 2.85 |  |  |
|  | 9mm | Ⅰ ^a^ | 2.95 | 1.61 | 1.82 | 2.71 | 4.00 | 7.231 | 0.027 ^K^* |
|  |  | Ⅱ ^ab^ | 2.55 | 1.41 | 1.59 | 2.37 | 3.18 |  |  |
|  |  | Ⅲ ^b^ | 2.44 | 1.27 | 1.55 | 2.14 | 3.16 |  |  |
| U6md | 5mm | Ⅰ ^a^ | 2.81 | 1.05 | 1.93 | 2.72 | 3.57 | 12.464 | 0.002 ^K^** |
|  |  | Ⅱ ^a^ | 2.70 | 1.08 | 1.92 | 2.63 | 3.19 |  |  |
|  |  | Ⅲ ^b^ | 2.33 | 0.93 | 1.68 | 2.22 | 2.91 |  |  |
|  | 7mm | Ⅰ ^a^ | 3.06 | 1.36 | 2.03 | 3.00 | 3.92 | 9.250 | 0.010 ^K^* |
|  |  | Ⅱ ^ab^ | 2.79 | 1.28 | 1.94 | 2.62 | 3.48 |  |  |
|  |  | Ⅲ ^b^ | 2.52 | 1.14 | 1.57 | 2.32 | 3.57 |  |  |
|  | 9mm | Ⅰ ^a^ | 3.72 | 1.86 | 2.31 | 3.39 | 5.01 | 10.796 | 0.005^K^** |
|  |  | Ⅱ ^ab^ | 3.31 | 1.62 | 2.01 | 3.03 | 4.19 |  |  |
|  |  | Ⅲ ^b^ | 2.94 | 1.50 | 1.69 | 2.52 | 4.15 |  |  |
| U67 | 5mm | Ⅰ ^a^ | 3.38 | 0.80 | 2.88 | 3.28 | 3.82 | 7.142 | 0.001 ^A^** |
|  |  | Ⅱ ^a^ | 3.42 | 0.81 | 2.85 | 3.40 | 3.93 |  |  |
|  |  | Ⅲ ^b^ | 3.04 | 0.91 | 2.43 | 2.93 | 3.66 |  |  |
|  | 7mm | Ⅰ | 3.75 | 0.98 | 3.12 | 3.80 | 4.37 | 2.530 | 0.081 ^A^ |
|  |  | Ⅱ | 3.66 | 0.99 | 3.11 | 3.50 | 4.26 |  |  |
|  |  | Ⅲ | 3.47 | 0.99 | 2.92 | 3.52 | 4.14 |  |  |
|  | 9mm | Ⅰ | 4.29 | 1.35 | 3.22 | 4.31 | 5.09 | 1.490 | 0.227^A^ |
|  |  | Ⅱ | 4.20 | 1.24 | 3.47 | 3.93 | 4.90 |  |  |
|  |  | Ⅲ | 4.00 | 1.23 | 3.16 | 3.99 | 4.86 |  |  |

SD: standard deviation; K, Kruskal-Wallis test; A, One way ANOVA analysis;

* Statistically significant at P <0 .05.

** Statistically significant at P <0 .01.

U56, between the maxillary second premolar and the first molar

U6md, between the mesiobuccal and distobuccal roots of the first molar

U67, between the first and second molar

^a b^ ^c^ statistically significant differences were observed between groups marked with the different letter.

**Table S9.** Comparison of buccal bone thickness (mm) among different sagittal faciall types.

| Area | Plane | Vertical facial type | Mean | SD | Percentiles | | | F/H | P |
| --- | --- | --- | --- | --- | --- | --- | --- | --- | --- |
|  |  |  |  |  | 25 | 50 | 75 |  |  |
| U56 | 5mm | hyperdivergent | 2.06 | 0.75 | 1.48 | 1.98 | 2.46 | 4.125 | 0.127^K^ |
|  |  | normodivergent | 2.30 | 1.00 | 1.48 | 2.12 | 3.08 |  |  |
|  |  | hypodivergent | 2.28 | 0.87 | 1.58 | 2.27 | 2.84 |  |  |
|  | 7mm | hyperdivergent | 2.17 | 0.89 | 1.52 | 2.09 | 2.69 | 2.524 | 0.283 ^K^ |
|  |  | normodivergent | 2.41 | 1.22 | 1.49 | 2.23 | 3.26 |  |  |
|  |  | hypodivergent | 2.40 | 1.03 | 1.58 | 2.24 | 3.20 |  |  |
|  | 9mm | Hyperdivergent ^a^ | 2.32 | 1.17 | 1.41 | 2.04 | 2.97 | 7.803 | 0.020^K^* |
|  |  | Normodivergent ^b^ | 2.86 | 1.65 | 1.58 | 2.69 | 3.81 |  |  |
|  |  | Hypodivergent ^a^ | 2.71 | 1.23 | 1.93 | 2.53 | 3.27 |  |  |
| U6md | 5mm | hyperdivergent | 2.48 | 0.87 | 1.78 | 2.51 | 2.91 | 4.086 | 0.130 ^K^ |
|  |  | normodivergent | 2.77 | 1.16 | 1.88 | 2.68 | 3.54 |  |  |
|  |  | hypodivergent | 2.59 | 0.94 | 1.84 | 2.47 | 3.20 |  |  |
|  | 7mm | hyperdivergent | 2.61 | 1.08 | 1.81 | 2.47 | 3.46 | 3.391 | 0.183 ^K^ |
|  |  | normodivergent | 2.95 | 1.45 | 1.94 | 2.82 | 3.88 |  |  |
|  |  | hypodivergent | 2.84 | 1.09 | 1.93 | 2.83 | 3.68 |  |  |
|  | 9mm | hyperdivergent | 3.02 | 1.44 | 1.96 | 2.67 | 4.10 | 5.320 | 0.070 ^K^ |
|  |  | normodivergent | 3.57 | 1.89 | 2.08 | 3.26 | 4.83 |  |  |
|  |  | hypodivergent | 3.38 | 1.46 | 2.15 | 3.13 | 4.49 |  |  |
| U67 | 5mm | hyperdivergent | 3.20 | 0.81 | 2.62 | 3.25 | 3.79 | 1.243 | 0.290^A^ |
|  |  | normodivergent | 3.36 | 0.88 | 2.74 | 3.27 | 3.83 |  |  |
|  |  | hypodivergent | 3.32 | 0.79 | 2.71 | 3.28 | 3.79 |  |  |
|  | 7mm | hyperdivergent | 3.62 | 0.95 | 2.99 | 3.60 | 4.25 | 0.697 | 0.498 ^A^ |
|  |  | normodivergent | 3.70 | 1.05 | 3.06 | 3.64 | 4.26 |  |  |
|  |  | hypodivergent | 3.56 | 0.86 | 2.93 | 3.54 | 4.23 |  |  |
|  | 9mm | hyperdivergent | 4.15 | 1.04 | 3.50 | 4.03 | 4.86 | 2.785 | 0.063 ^A^ |
|  |  | normodivergent | 4.30 | 1.39 | 3.29 | 4.14 | 5.13 |  |  |
|  |  | hypodivergent | 3.87 | 1.29 | 3.02 | 3.64 | 4.83 |  |  |

SD: standard deviation; K, Kruskal-Wallis test; A, One way ANOVA analysis;

* Statistically significant at P <0 .05.

U56, between the maxillary second premolar and the first molar

U6md, between the mesiobuccal and distobuccal roots of the first molar

U67, between the first and second molar

^a b^ statistically significant differences were observed between groups marked with the different letter.

**Table S10. Buccal interdental root distances (mm) distribution at different planes.**

| Plane | Area | Mean | SD | Percentiles | | | F/H | P |
| --- | --- | --- | --- | --- | --- | --- | --- | --- |
|  |  |  |  | 25 | 50 | 75 |  |  |
| 5mm | U56 ^a^ | 3.52 | 0.98 | 2.83 | 3.56 | 4.22 | 220.68 | <0.001^K^*** |
|  | U6md ^b^ | 1.70 | 0.59 | 1.29 | 1.68 | 2.08 |  |  |
|  | U67 ^c^ | 2.00 | 1.08 | 1.27 | 1.83 | 2.58 |  |  |
| 7mm | U56 ^a^ | 4.07 | 1.15 | 3.22 | 4.10 | 4.88 | 513.46 | <0.001^K^*** |
|  | U6md ^b^ | 1.98 | 0.74 | 1.46 | 1.92 | 2.46 |  |  |
|  | U67 ^b^ | 2.19 | 1.26 | 1.23 | 1.97 | 2.92 |  |  |
| 9mm | U56 ^a^ | 5.01 | 1.44 | 4.00 | 5.02 | 5.95 | 499.89 | <0.001^K^*** |
|  | U6md ^b^ | 2.10 | 1.05 | 1.26 | 1.97 | 2.71 |  |  |
|  | U67 ^c^ | 2.84 | 1.52 | 1.67 | 2.64 | 3.86 |  |  |

SD: standard deviation; Kruskal-Wallis test;

*** Statistically significant at P <0 .001.

U56, between the maxillary second premolar and the first molar

U6md, between the mesiobuccal and distobuccal roots of the first molar

U67, between the first and second molar

^a b^ ^c^ statistically significant differences were observed between groups marked with the different letter.

**Table S11.** Comparison of buccal interdental root distances (mm) of different genders.

| Area | Plane | Male | | Female | | t/Z | P |
| --- | --- | --- | --- | --- | --- | --- | --- |
|  |  | Mean | SD | Mean | SD |  |  |
| U56 | 5mm | 3.66 | 1.00 | 3.46 | 0.97 | -1.836 | 0.067^T^ |
|  | 7mm | 4.05 | 1.17 | 4.09 | 1.14 | 0.316 | 0.752 ^T^ |
|  | 9mm | 4.90 | 1.32 | 5.05 | 1.49 | 0.956 | 0.340 ^T^ |
| U6md | 5mm | 1.81 | 0.55 | 1.66 | 0.61 | -2.221 | 0.027 ^T^* |
|  | 7mm | 2.26 | 0.70 | 1.87 | 0.73 | -4.764 | <0.001^T^*** |
|  | 9mm | 2.44 | 1.01 | 1.95 | 1.03 | -4.502 | <0.001^U^*** |
| U67 | 5mm | 1.82 | 0.96 | 2.07 | 1.12 | -2.031 | 0.042 ^U^* |
|  | 7mm | 1.96 | 1.21 | 2.28 | 1.26 | -2.530 | 0.011 ^U^* |
|  | 9mm | 2.43 | 1.33 | 3.01 | 1.57 | -3.324 | 0.001^U^** |

SD: standard deviation; U, Mann-Whitney U test; T, Independent sample t test;

* Statistically significant at P <0 .05.

** Statistically significant at P <0 .01.

*** Statistically significant at P <0 .001.

U56, between the maxillary second premolar and the first molar

U6md, between the mesiobuccal and distobuccal roots of the first molar

U67, between the first and second molar

**Table S12.** Comparison of buccal interdental root distances (mm) among different sagittal skeletal types.

| Area | Plane | Sagittal skeletal type | Mean | SD |  | | | F/H | P |
| --- | --- | --- | --- | --- | --- | --- | --- | --- | --- |
|  |  |  |  |  | 25 | 50 | 75 |  |  |
| U56 | 5mm | Ⅰ | 3.63 | 0.95 | 2.93 | 3.65 | 4.39 | 2.03 | 0.132^A^ |
|  |  | Ⅱ | 3.44 | 1.00 | 2.77 | 3.42 | 4.17 |  |  |
|  |  | Ⅲ | 3.42 | 1.00 | 2.64 | 3.34 | 4.09 |  |  |
|  | 7mm | Ⅰ | 4.23 | 1.14 | 3.43 | 4.18 | 5.09 | 2.63 | 0.073^A^ |
|  |  | Ⅱ | 3.99 | 1.13 | 3.25 | 4.07 | 4.74 |  |  |
|  |  | Ⅲ | 3.94 | 1.18 | 2.96 | 3.93 | 4.98 |  |  |
|  | 9mm | Ⅰ | 5.17 | 1.38 | 4.22 | 5.12 | 6.02 | 2.23 | 0.109^A^ |
|  |  | Ⅱ | 4.99 | 1.48 | 4.00 | 5.02 | 6.12 |  |  |
|  |  | Ⅲ | 4.77 | 1.47 | 3.68 | 4.69 | 5.75 |  |  |
| U6md | 5mm | Ⅰ ^a^ | 1.78 | 0.60 | 1.37 | 1.74 | 2.21 | 6.91 | 0.001^A^** |
|  |  | Ⅱ ^a^ | 1.74 | 0.59 | 1.30 | 1.70 | 2.09 |  |  |
|  |  | Ⅲ ^b^ | 1.51 | 0.54 | 1.16 | 1.50 | 1.85 |  |  |
|  | 7mm | Ⅰ ^a^ | 2.06 | 0.75 | 1.58 | 2.01 | 2.59 | 3.67 | 0.026^A^* |
|  |  | Ⅱ ^ab^ | 2.01 | 0.75 | 1.43 | 1.91 | 2.53 |  |  |
|  |  | Ⅲ ^b^ | 1.81 | 0.69 | 1.35 | 1.77 | 2.24 |  |  |
|  | 9mm | Ⅰ | 2.21 | 1.16 | 1.35 | 2.05 | 2.81 | 4.35 | 0.114^K^ |
|  |  | Ⅱ | 2.12 | 0.97 | 1.38 | 1.97 | 2.83 |  |  |
|  |  | Ⅲ | 1.88 | 0.94 | 1.15 | 1.83 | 2.60 |  |  |
| U67 | 5mm | Ⅰ | 1.87 | 0.98 | 1.22 | 1.64 | 2.49 | 5.72 | 0.057^K^ |
|  |  | Ⅱ | 2.00 | 1.04 | 1.16 | 1.90 | 2.61 |  |  |
|  |  | Ⅲ | 2.20 | 1.27 | 1.43 | 2.05 | 2.79 |  |  |
|  | 7mm | Ⅰ ^a^ | 2.08 | 1.28 | 1.09 | 1.65 | 2.79 | 6.07 | 0.048^K^* |
|  |  | Ⅱ ^ab^ | 2.14 | 1.17 | 1.18 | 2.00 | 2.78 |  |  |
|  |  | Ⅲ ^b^ | 2.43 | 1.30 | 1.40 | 2.24 | 3.12 |  |  |
|  | 9mm | Ⅰ ^a^ | 2.59 | 1.46 | 1.45 | 2.42 | 3.67 | 8.61 | 0.013^K^* |
|  |  | Ⅱ ^ab^ | 2.87 | 1.55 | 1.62 | 2.64 | 3.88 |  |  |
|  |  | Ⅲ ^b^ | 3.18 | 1.51 | 2.05 | 2.99 | 4.23 |  |  |

SD: standard deviation; K, Kruskal-Wallis test; A, One way ANOVA analysis;

* Statistically significant at P <0 .05.

**Statistically significant at P <0 .01.

U56, between the maxillary second premolar and the first molar

U6md, between the mesiobuccal and distobuccal roots of the first molar

U67, between the first and second molar

^a b^ statistically significant differences were observed between groups marked with the different letter.

**Table S13.** Comparison of buccal interdental root distances (mm) among different vertical facial types.

| Area | Plane | Vertical facial type | Mean | SD | Percentiles | | | F/H | P |
| --- | --- | --- | --- | --- | --- | --- | --- | --- | --- |
|  |  |  |  |  | 25 | 50 | 75 |  |  |
| U56 | 5mm | hyperdivergent ^a^ | 3.35 | 1.06 | 2.50 | 3.35 | 4.14 | 3.92 | 0.021^A^* |
|  |  | normodivergent ^ab^ | 3.52 | 0.95 | 2.79 | 3.57 | 4.22 |  |  |
|  |  | hypodivergent ^b^ | 3.75 | 0.93 | 3.02 | 3.81 | 4.37 |  |  |
|  | 7mm | hyperdivergent ^a^ | 3.89 | 1.20 | 3.07 | 4.08 | 4.83 | 5.00 | 0.007^A^** |
|  |  | normodivergent ^a^ | 4.04 | 1.14 | 3.12 | 3.94 | 4.87 |  |  |
|  |  | hypodivergent ^b^ | 4.42 | 1.04 | 3.58 | 4.29 | 5.21 |  |  |
|  | 9mm | hyperdivergent | 4.90 | 1.54 | 3.85 | 5.03 | 6.01 | 0.57 | 0.567^A^ |
|  |  | normodivergent | 5.02 | 1.45 | 3.90 | 4.95 | 6.05 |  |  |
|  |  | hypodivergent | 5.14 | 1.26 | 4.33 | 5.05 | 5.75 |  |  |
| U6md | 5mm | hyperdivergent | 1.62 | 0.61 | 1.23 | 1.61 | 1.96 | 1.60 | 0.204^A^ |
|  |  | normodivergent | 1.72 | 0.57 | 1.36 | 1.68 | 2.09 |  |  |
|  |  | hypodivergent | 1.77 | 0.62 | 1.27 | 1.77 | 2.20 |  |  |
|  | 7mm | hyperdivergent | 1.96 | 0.79 | 1.39 | 1.85 | 2.52 | 0.79 | 0.453^A^ |
|  |  | normodivergent | 1.96 | 0.71 | 1.52 | 1.93 | 2.42 |  |  |
|  |  | hypodivergent | 2.08 | 0.75 | 1.53 | 2.01 | 2.64 |  |  |
|  | 9mm | hyperdivergent | 2.03 | 1.00 | 1.23 | 1.93 | 2.73 | 5.95 | 0.051^K^ |
|  |  | normodivergent | 2.02 | 0.99 | 1.22 | 1.89 | 2.63 |  |  |
|  |  | hypodivergent | 2.43 | 1.22 | 1.70 | 2.17 | 2.82 |  |  |
| U67 | 5mm | hyperdivergent | 2.03 | 1.23 | 1.40 | 1.83 | 2.62 | 1.00 | 0.608^K^ |
|  |  | normodivergent | 1.96 | 1.06 | 1.23 | 1.78 | 2.56 |  |  |
|  |  | hypodivergent | 2.04 | 0.94 | 1.29 | 2.00 | 2.64 |  |  |
|  | 7mm | hyperdivergent | 1.96 | 0.79 | 1.24 | 1.90 | 2.99 | 1.42 | 0.493^K^ |
|  |  | normodivergent | 1.96 | 0.71 | 1.19 | 1.86 | 2.80 |  |  |
|  |  | hypodivergent | 2.08 | 0.75 | 1.21 | 2.13 | 3.12 |  |  |
|  | 9mm | hyperdivergent | 2.95 | 1.55 | 1.72 | 2.56 | 3.99 | 0.61 | 0.737^K^ |
|  |  | normodivergent | 2.80 | 1.57 | 1.57 | 2.67 | 3.83 |  |  |
|  |  | hypodivergent | 2.78 | 1.33 | 1.85 | 2.76 | 3.84 |  |  |

SD: standard deviation; K, Kruskal-Wallis test; A, One way ANOVA analysis;

* Statistically significant at P <0.05.

** Statistically significant at P <0.01

U56, between the maxillary second premolar and the first molar

U6md, between the mesiobuccal and distobuccal roots of the first molar

U67, between the first and second molar

^a b^ statistically significant differences were observed between groups marked with the different letter.

**Table S14**. Comparison of maxillary retromolar space (mm) of different genders.

| Plane | Male | | Female | | t/Z | P |
| --- | --- | --- | --- | --- | --- | --- |
|  | Mean | SD | Mean | SD |  |  |
| 5mm | 9.30 | 2.76 | 9.34 | 2.68 | -0.41 | 0.680^U^ |
| 7mm | 10.49 | 2.63 | 10.21 | 2.46 | -1.44 | 0.153^U^ |
| 9mm | 11.39 | 2.47 | 10.95 | 2.38 | -1.62 | 0.107^T^ |

SD: standard deviation; U, Mann-Whitney U test; T, Independent sample t test;

**Table S15**. Comparison of maxillary retromolar space (mm) of different sagittal skeletal types.

| Plane | Sagittal skeletal type | Mean | SD | Percentiles | | | F/H | P |
| --- | --- | --- | --- | --- | --- | --- | --- | --- |
|  |  |  |  | 25 | 50 | 75 |  |  |
| 5mm | Ⅰ ^a^ | 8.86 | 3.02 | 6.25 | 9.53 | 11.03 | 6.52 | 0.038^K^* |
|  | Ⅱ ^b^ | 9.90 | 2.42 | 8.04 | 9.85 | 11.30 |  |  |
|  | Ⅲ ^ab^ | 9.33 | 2.36 | 7.86 | 9.47 | 11.26 |  |  |
| 7mm | Ⅰ | 9.90 | 2.82 | 8.01 | 10.39 | 11.93 | 4.51 | 0.105^K^ |
|  | Ⅱ | 10.73 | 2.26 | 9.42 | 10.75 | 11.96 |  |  |
|  | Ⅲ | 10.34 | 2.19 | 8.50 | 10.34 | 11.93 |  |  |
| 9mm | Ⅰ ^a^ | 10.71 | 2.51 | 9.20 | 10.83 | 12.49 | 3.59 | 0.029^A^* |
|  | Ⅱ ^b^ | 11.46 | 2.32 | 10.31 | 11.44 | 12.71 |  |  |
|  | Ⅲ ^ab^ | 11.15 | 2.33 | 9.41 | 11.42 | 12.61 |  |  |

SD: standard deviation; K, Kruskal-Wallis test; A, One way ANOVA analysis;

* Statistically significant at P <0 .05.

^a b^ statistically significant differences were observed between groups marked with the different letter.

**Table S16**. Comparison of maxillary retromolar space (mm) of different vertical facial types.

| Plane | Vertical facial type | Mean | SD | Percentiles | | | F/H | P |
| --- | --- | --- | --- | --- | --- | --- | --- | --- |
|  |  |  |  | 25 | 50 | 75 |  |  |
| 5mm | hyperdivergent | 9.40 | 2.69 | 7.58 | 9.43 | 11.17 | 0.118 | 0.943 ^K^ |
|  | normodivergent | 9.31 | 2.82 | 7.41 | 9.77 | 11.30 |  |  |
|  | hypodivergent | 9.29 | 2.41 | 7.59 | 9.55 | 11.22 |  |  |
| 7mm | hyperdivergent | 10.26 | 2.55 | 8.53 | 10.30 | 11.97 | 0.298 | 0.862 ^K^ |
|  | normodivergent | 10.30 | 2.63 | 8.64 | 10.60 | 12.01 |  |  |
|  | hypodivergent | 10.31 | 2.12 | 9.08 | 10.38 | 11.70 |  |  |
| 9mm | hyperdivergent | 11.09 | 2.59 | 9.35 | 10.93 | 12.71 | 0.025 | 0.975 ^A^ |
|  | normodivergent | 11.05 | 2.49 | 9.51 | 11.36 | 12.65 |  |  |
|  | hypodivergent | 11.12 | 1.90 | 9.96 | 11.20 | 12.40 |  |  |

SD: standard deviation; K, Kruskal-Wallis test; A, One way ANOVA analysis;

**Table S17**. The influence of the existence of the maxillary third molar on the maxillary retromolar space (mm).

| Plane | Exist | | Do not exist | | t/Z | P |
| --- | --- | --- | --- | --- | --- | --- |
|  | Mean | SD | Mean | SD |  |  |
| 5mm | 9.45 | 2.68 | 8.98 | 2.73 | -1.341 | 0.180^U^ |
| 7mm | 10.50 | 2.42 | 9.67 | 2.66 | -2.270 | 0.023^U^* |
| 9mm | 11.39 | 2.21 | 10.16 | 2.74 | -4.043 | <0.001^T^*** |

SD: standard deviation; U, Mann-Whitney U test; T, Independent sample t test;

* Statistically significant at P <0 .05.

*** Statistically significant at P <0 .001.
